# Theoretical Estimate of the Glass Transition Line of Yukawa One-Component Plasmas

**DOI:** 10.3390/molecules26030669

**Published:** 2021-01-28

**Authors:** Federico Lucco Castello, Panagiotis Tolias

**Affiliations:** Space and Plasma Physics, Royal Institute of Technology, SE-100 44 Stockholm, Sweden; tolias@kth.se

**Keywords:** glass transition, mode coupling theory, integral equation theory, Yukawa one-component plasmas, isomorph theory, quasi-universality

## Abstract

The mode coupling theory of supercooled liquids is combined with advanced closures to the integral equation theory of liquids in order to estimate the glass transition line of Yukawa one-component plasmas from the unscreened Coulomb limit up to the strong screening regime. The present predictions constitute a major improvement over the current literature predictions. The calculations confirm the validity of an existing analytical parameterization of the glass transition line. It is verified that the glass transition line is an approximate isomorphic curve and the value of the corresponding reduced excess entropy is estimated. Capitalizing on the isomorphic nature of the glass transition line, two structural vitrification indicators are identified that allow a rough estimate of the glass transition point only through simple curve metrics of the static properties of supercooled liquids. The vitrification indicators are demonstrated to be quasi-universal by an investigation of hard sphere and inverse power law supercooled liquids. The straightforward extension of the present results to bi-Yukawa systems is also discussed.

## 1. Introduction

When liquids are quenched below their melting point by cooling or compression in a manner that suppresses crystallization [1], they exhibit a dramatic slowdown in dynamics and remarkable increase in their viscosity. Since quenching is typically caused by cooling, these metastable liquids are known as supercooled and, for a sufficiently low temperature, they can undergo dynamical arrest and transform into a glass [2]. The process of liquid-glass transition or more simply glass transition has been the source of various questions concerning the nature of the transition and the microscopic mechanisms driving it [3,4,5].

The physics of the glass transition have been addressed with a mix of experiments, computer simulations and theoretical approaches. In experiments, the glass transition has been investigated in colloidal systems [6,7,8], granular media [9,10] and organic compounds [11]. Simulations have led to important insight in the physics of supercooled liquids in regimes often not accessible in experiments by adopting simplified models such as the Kob–Andersen [12,13,14] and hard-sphere binary mixtures [15,16]. Theoretical approaches such as mode coupling theory [17,18], random first-order transition theory [19] and dynamic facilitation theory [20] have rationalized some experimental findings and even predicted previously unobserved features of the vitrification process [21,22].

In this paper, mode coupling theory (MCT) is employed to estimate the glass transition line of Yukawa one-component plasmas (YOCP). MCT is an entirely first-principle approach for the investigation of the dynamic processes occurring in glass-forming liquids, which allows to localize the glass transition point requiring only the knowledge of the static properties of the supercooled liquid as an input. MCT is known to lead to good predictions for the dynamics of supercooled liquids [22], which also applies for the glass transition point in spite of the fact that the MCT bifurcation (associated with the glass transition) should rather be interpreted as a cross-over point from a non-activated into an activated dynamics regime [4,22]. The YOCP comprises of equally charged point particles immersed in a neutralizing background that interact via the pair potential $u\left(r\right)=\left(Q^{2}/r\right)\exp\left(-r/\lambda \right)$. Here *Q* is the particle charge and λ the screening length defined by the polarizable background. Thermodynamic YOCP states are uniquely specified by two dimensionless variables [23]: the coupling parameter $\Gamma =\beta Q^{2}/d$ and the screening parameter κ=λ/d. This allows to re-write the interaction potential as βu(x)=(Γ/x)exp(−κx), where $d={\left(4\pi n/3\right)}^{1/3}$ is the Wigner–Seitz radius, *n* is the particle number density, $\beta =1/(k_{B}T)$ and x=r/d is a normalized distance. The YOCP possesses a well-understood phase diagram in terms of the κ and Γ variables [24].

The main motivation of this work lies in the relevance of the YOCP model to the experimental realization of complex plasmas, a novel state of soft matter composed of charged particles of mesoscopic size that are immersed in a weakly ionized plasma [23]. The YOCP has been suggested as a promising tool to investigate the dynamics of glassy systems [25], but glass formation has remained experimentally elusive in three dimensional complex plasmas. In particular, complex plasmas have already been employed in order to study supercooled fluids near the vitrification point in two dimensions [26,27], but three-dimensional glassy structures exhibiting dynamical arrest still remain to be observed. The accurate estimate of the glass transition line provided in this work should help to guide current [28] or future complex plasma experiments in microgravity conditions that are or will be actively searching for the glassy state of plasmas. It is worth pointing out that MCT calculations of the YOCP glass transition line are already available in the literature [29]. However, there is space for drastic improvement over the existing prediction due to the use of oversimplified structural input that should be grossly inaccurate within the supercooled liquid regime.

## 2. Theoretical Background

This section provides an overview of the theoretical background upon which the remaining part of this work is constructed. The equations and approximations which characterize mode coupling theory are presented, the basics of isomorph theory are discussed and the integral equation theory of liquids employed to compute the static structural properties is presented.

### 2.1. Mode Coupling Theory of the Glass Transition

In order to distinguish glasses from stable and supercooled fluids, it is necessary to consider the temporal evolution of the microscopic dynamics. One common probe for such dynamics is the intermediate scattering function, F(k,t), which quantifies, at time *t* and for the wavenumber *k*, the correlation of the density fluctuations over a length scale ∼$k^{-1}$. The temporal dependence of F(k,t) exhibits three distinguished behaviors between the fluid, supercooled and glassy state [30]: for stable fluids, the intermediate scattering function relaxes exponentially in time, $F\left(k,t\right)∼e^{-t/\tau }$. On the other hand, supercooled liquids exhibit a multi-stage relaxation in which an initial exponential decay is followed by the so-called β-relaxation regime in which the particles are trapped in cages and the intermediate scattering function exhibits a plateau, i.e., F(k,t)≈const.r Such plateau is eventually destroyed during the final α-relaxation regime when the particles escape from their cages, ergodicity is restored and the intermediate scattering function relaxes towards zero following a stretched-exponential law, $F\left(k,t\right)∼e^{-(t/\tau )\gamma }$. In the above τ and γ are two constants which are both wavenumber and temperature dependent. Finally, for glasses, there is no full relaxation of the dynamics, particles escape their cages only in rare events and the intermediate scattering function is characterized by a persistent plateau which leads to a positive asymptotic limit F(k,t→∞)>0.

MCT provides an equation of motion for the intermediate scattering function [17,18]. In what follows, we will briefly describe the derivation of the fundamental MCT equation with the main purpose of highlighting the assumptions that are adopted in MCT, the reader is addressed to Ref. [30] for a detailed derivation of the MCT equation of motion. Let us start by considering a system with *N* particles of mass *m* that are enclosed in a volume *V* with average number density n=N/V and temperature *T*. Using r${}_{j}\left(t\right)$ to denote the position of a particle *j* at a time *t*, we introduce the time-dependent microscopic density $\rho \left(r,t\right)=\sum_{j=1}^{N}\delta \left[r-r_{j}\left(t\right)\right]$ and the microscopic density fluctuations δρ(r,t)=n−ρ(r,t). The intermediate scattering function is then defined as the autocorrelation function of the density fluctuations, F(k,t)=(1/N)〈δρ(−k,0)δρ(k,t)〉, where 〈…〉 denotes the ensemble average operator and where $\delta \rho \left(k,t\right)=\sum_{j=1}^{N}\exp\left[ik\cdot r_{j}\left(t\right)\right]-{\left(2\pi \right)}^{3}n\delta$(k) is the spatial Fourier transform of the density fluctuations (we have implicitly assumed that the system is isotropic). Employing the Zwanzig–Mori projection formalism [31,32], it is possible to obtain the following exact integro-differential equation for the intermediate scattering function [18]

(1) $$\frac{\partial^{2}F\left(k,t\right)}{\partial t^{2}}+{\left[\Omega \left(k\right)\right]}^{2}F\left(k,t\right)+\int_{0}^{t}dt^{\prime }M\left(k,t^{\prime }\right)\frac{\partial F(k,t-t^{\prime })}{\partial t}=0$$

where ${\left[\Omega \left(k\right)\right]}^{2}=k^{2}/\left[\beta mS\left(k\right)\right]$, S(k)=F(k,0) is the static structure factor and M(k,t) is a memory kernel which can be expressed as the ensemble average of a fluctuating force that depends on density fluctuations and pair density products of the form δρ(−k,t)δρ(k,t) [30]. Equation (1) has the structure of an oscillator equation in which Ω(k) plays the role of the characteristic frequency, while the memory kernel acts as a generalized and time-dependent friction coefficient. However, without a simplified expression for the memory function, Equation (1) cannot be solved. Within MCT, such simplified expression for M(k,t) is derived by adopting the procedure explained below.

In MCT, the memory kernel is decomposed into the regular $M_{\mathrm{reg}}\left(k,t\right)$ part that describes the short-time conventional liquid dynamics and the asymptotic $M_{\mathrm{MCT}}\left(k,t\right)$ part that describes the long-time dynamics dominated by the interplay between caging and ergodicity-restoring effects [18], $M\left(k,t\right)=M_{\mathrm{reg}}\left(k,t\right)+M_{\mathrm{MCT}}\left(k,t\right)$. The regular part is neglected [30] or approximated with $M_{\mathrm{reg}}\left(k,t\right)=\nu \delta \left(t\right)$ with ν a friction constant [17,33], since the glassy state is mainly concerned with the long-time behavior of the density correlation function. The second part is treated under the assumption that the most relevant contribution stems from the pair density products of the fluctuating force. Hence, $M_{\mathrm{MCT}}\left(k,t\right)$ is projected onto a basis of pair density products $\rho \left(k_{1},t\right)\rho \left(k_{2},t\right)$ with a properly defined projection operator running over all $\left(k_{1},k_{2}\right)$ pairs relevant to the system [30]. The projection leads to the emergence of triplet correlation functions containing only static properties and of a time-dependent four particle density correlation function; the triplet correlation functions are factorized into static structure factor products within the convolution approximation [34], while the four particle density correlation function is approximated as the product of density pair correlation functions with Kawasaki’s approach [35]. These lead to the MCT intermediate scattering function equation

(2) $$\tau \left(k\right)\left[\frac{1}{\nu }\frac{\partial^{2}F\left(k,t\right)}{\partial t^{2}}+\frac{\partial F(k,t)}{\partial t}\right]+F\left(k,t\right)+\int_{0}^{t}dt^{\prime }M_{\mathrm{MCT}}\left(k,t^{\prime }\right)\frac{\partial F(k,t-t^{\prime })}{\partial t}=0$$

where $\tau \left(\kappa \right)=\nu /{\left[\Omega \left(k\right)\right]}^{2}$ and the memory kernel is given by [30]

(3) $$M_{\mathrm{MCT}}\left(k,t\right)=\frac{nS(k)}{16\pi^{3}k^{4}}\int d^{3}k^{\prime }F\left(k^{\prime },t\right)F\left(p,t\right){\left[\left(k\cdot k^{\prime }\right)c\left(k^{\prime }\right)+\left(k\cdot p\right)c\left(p\right)\right]}^{2}.$$

In the above p=|p|, $p=k-k^{\prime }$ and c(k) is the Fourier transform of the direct correlation function which is related to the static structure factor via the Ornstein–Zernike equation (see Section 2.3), S(k)=1/[1−nc(k)]. Provided that the static structure factor and the viscosity are known, Equation (2) is a self-consistent equation for F(k,t) which can be solved subject to the initial conditions F(k,0)=S(k) and ∂F(k,0)/∂t=0. Equation (2) is sometimes reported in its over-damped form by assuming that the viscosity is so large that the inertial second order derivative term can be neglected [18,33,36]. However, while the over-damped form of Equation (2) is appropriate to study colloids, it would be inaccurate for complex plasmas in which the dilute background gas [23] makes viscous damping less predominant.

Given that glasses can be distinguished from conventional liquids from the asymptotic limit of the intermediate scattering function, it is sufficient to obtain an equation for F(k,t→∞) in order to investigate the glass transition properties. Rewriting Equation (2) in terms of the normalized density autocorrelation function ϕ(k,t)=F(k,t)/S(k), Laplace transforming and employing the final value theorem to extract the asymptotic limit, leads to the form factor $f\left(k\right)=\lim_{t\to \infty }\phi \left(k,t\right)$ MCT equation

(4) $$\frac{f(k)}{1-f(k)}=\frac{nS(k)}{16\pi^{3}k^{4}}\int d^{3}k^{\prime }S\left(k^{\prime }\right)S\left(p\right){\left[{\left(k\cdot k\right)}^{\prime }c\left(k^{\prime }\right)+\left(k\cdot p\right)c\left(p\right)\right]}^{2}f\left(k^{\prime }\right)f\left(p\right).$$

The solution of the MCT equation for the form factor allows to distinguish between liquids and glasses, since the former are characterized by f(k)=0 while the latter are characterized by f(k)>0. It should be noted that, at the glass transition point, the form factor changes discontinuously from zero to some positive critical value $f_{c}\left(k\right)>0$. This discontinuity in the form factor happens despite the fact that the static properties do not exhibit any discontinuity between supercooled liquids and glasses. This bifurcation phenomenon is a manifestation of the feedback between the force fluctuations in the memory kernel and the density fluctuations in the form factor [18,21].

### 2.2. Isomorph Theory

Isomorphic curves are phase diagram lines of constant excess entropy along which a substantial set of structural and dynamical properties remain approximately invariant when expressed in dimensionless units where the length is normalized to $a=n^{1/3}$ and the energy to $k_{B}T$ [37,38]. While it is possible to identify lines of constant excess entropy in the phase diagram of any system, only in R-simple systems the isentropic lines are also isomorphs. R-simple systems are characterized by the property that the ordering of the potential energies of two configurations corresponding to the same density is preserved when the two configurations are re-scaled uniformly to a different density [39]. In other words, denoting with *U* the potential energy and with R the set of N particle’s position $\left\{r_{1},\ldots r_{N}\right\}$, R-simple systems satisfy the relation $U\left(R_{a}\right)<U\left(R_{b}\right)\Rightarrow U\left(\zeta R_{a}\right)<U\left(\zeta R_{b}\right)$, where $R_{a}$ and $R_{b}$ are two equal-density configurations and ζ a positive scaling factor.

A recent investigation has shown that the YOCP is an R-simple system whose isomorphs can be accurately described with the following analytical parameterization [40]

(5) $$\Gamma_{\mathrm{iso}}\left(\Gamma ,\kappa \right)=\Gamma e^{-\Lambda \alpha \kappa }\left[1+\left(\Lambda \alpha \kappa \right)+\frac{{\left(\Lambda \alpha \kappa \right)}^{2}}{2}\right]=\mathrm{const},$$

where $\alpha =a/d={\left(4\pi /3\right)}^{1/3}$ is the ratio between the mean-cubic inter-particle distance employed in isomorph theory and the Wigner–Seitz radius used for the characterization of the YOCP state point, while Λ is a constant close to unity which depends weakly on the state point. It should be noted that, with the assumption Λ=1, the isomorph parameterization described by Equation (5) is equivalent to the semi-empirical expression utilized in Ref. [41] in order to fit MD data of the YOCP melting line [24,42]

(6) $$\Gamma_{m}\left(\kappa \right)=\Gamma_{m}^{\mathrm{OCP}}e^{\alpha \kappa }{\left[1+\alpha \kappa +\frac{{\left(\alpha \kappa \right)}^{2}}{2}\right]}^{-1},$$

where $\Gamma_{m}^{\mathrm{OCP}}=171.8$ is the coupling parameter at melting in the one-component plasma (OCP) limit (κ=0) [42]. The fact that the isomorphs and the melting line can be approximated with the same analytical expression is a reflection of the fact that for R-simple systems the melting line constitutes an isomorphic curve to the first-order [37,43].

### 2.3. Integral Equation Theory of Liquids

The integral equation theory (IET) of liquids gives access to the static liquid properties without resorting to computer simulations. For one-component liquids with pair-wise isotropic interactions, IET comprises of two exact equations: the Ornstein–Zernike equation [34]

(7) $$h\left(r\right)=c\left(r\right)+n\int c\left(r^{\prime }\right)h(|r-r^{\prime }\left|\right)d^{3}r^{\prime }\;$$

and the non-linear closure condition derived from cluster diagram analysis [34]

(8) $$g\left(r\right)=\exp\left[-\beta u(r)+h(r)-c(r)+B(r)\right].$$

In the above g(r) denotes the radial distribution function, h(r)=g(r)−1 the total correlation function, c(r) the direct correlation function and B(r) the bridge function. Knowledge of the total correlation function h(r) gives direct access to the static structure factor via the Fourier space relation S(k)=1+nh(k). Thus, the solution of the above equations provides all the input that is necessary for the calculation of the form factor via MCT. Nevertheless, the solution of the IET system of equations requires an expression for the bridge function. The latter can be formally represented as a power series of the density, but such series is known to converge slowly already at moderate densities [44,45]. Combined with the fact that the calculation of the series coefficients becomes extremely cumbersome beyond the third order [46,47,48], this calls for the adoption of approximate bridge function expressions.

Among the wide variety of bridge function approximations or closures that have been proposed over the years [49], here we shall consider only three approximations which have been previously employed to investigate the fluid properties of the YOCP: the hypernetted chain (HNC) approximation, the isomorph-based empirically-modified hypernetted chain (IEMHNC) approach and the variational modified hypernetted chain (VMHNC) approximation. Within the HNC approximation, the bridge function is neglected altogether by assuming B(r)=0 [50]. The HNC approximation is straightforward to implement and flexible, since it is not taylored to any specific interaction potential. For systems featuring Coulomb interactions, it has been shown that the HNC approximation produces qualitatively correct results for the static and thermodynamic properties of state points far from crystallization, but that its performance rapidly degrades near the freezing line [51].

The IEMHNC approach is an advanced closure to the IET system of equations that is built upon the ansatz that reduced-unit bridge functions remain exactly invariant along isomorphic lines [52]. Systematic indirect bridge function extractions along isomorphic curves with the aid of molecular dynamics simulations have confirmed the approximate validity of the invariance conjecture for Yukawa systems [53]. This ansatz is then combined with two external inputs, a closed-form expression for the isomorphs and a closed-form bridge function expression valid along any phase diagram line that possesses a unique intersection point with any isomorph, in order to construct an expression for the bridge function valid for the whole phase diagram [52]. The IEMHNC approach has been applied to Yukawa and bi-Yukawa fluids showing a remarkable agreement with computer simulations in the entire dense fluid region up to crystallization [52,54]. Note that the IEMHNC approach is only applicable to R-simple systems for which the aforementioned external input is available. In addition, the IEMHNC bridge function expression might be accurate only in a sub-region of the phase diagram which depends on the region of validity of the external inputs. For instance, with the currently available external inputs, the IEMHNC bridge function for the YOCP is strictly applicable for coupling parameters which satisfy $5.25\leq \Gamma_{\mathrm{iso}}\left(\Gamma ,\kappa \right)\leq 171.8$ [52].

The VMHNC approximation is an advanced closure to the IET system of equations which is based on the ansatz of bridge function quasi-universality [55]. The quasi-universality conjecture justifies the following two-step procedure adopted to define the bridge function [56]: first, the unknown bridge function B(r) is replaced with the Percus–Yevick bridge function of the hard sphere system, $B_{\mathrm{HSPY}}\left(r;\eta \right)$, for which an exact analytical representation in terms of the inter-particle separation *r* and of the packing fraction $\eta =\pi n\sigma^{3}/6$ (with σ the hard sphere diameter) is available. Second, the value of the packing fraction which ensures the correct mapping between B(r) and $B_{\mathrm{HSPY}}\left(r;\eta \right)$ is determined with a robust method based on the minimization of a properly defined free energy functional. Since it is not constructed for a specific class of systems, the VMHNC approach shares the same flexibility of the HNC approximation and can be applied without any major modification to any one-component system characterized by purely repulsive interactions. For the specific case of the YOCP, the VMHNC is known to produce results that compare exceptionally well with computer simulations [57] and are as accurate as those obtained with the IEMHNC approach over the entire fluid region of the phase diagram [58]. The main drawback of the VMHNC approach lies in its computational cost, since the minimization of the free energy functional makes the algorithm for the solution of the IET more cumbersome, eventually causing the VMHNC approach to become up to 80 times slower than approximations which do not involve minimization [58].

## 3. Computational Approach

This section describes the computational methods employed in the solution of the MCT equation for the form factor and in the solution of the IET system of equations for the static structural properties. The advantages of combining MCT with structural input obtained with advanced IET closures are discussed and the present algorithm is benchmarked against the literature results.

### 3.1. Combining MCT with Advanced IET Approaches

Within MCT, the static structural properties of the supercooled fluid constitute the only external to the theory input that is required for the calculation of the glass transition line and the critical form factors. In the literature, such properties have been obtained either from computer simulations [59,60,61] or from IET calculations combined with elementary closures (when simulation input was unavailable) which include the aforementioned HNC approximation for soft long range interaction potentials [29,62] or the Percus–Yevick (PY) approximation for hard sphere short range interactions [16,17,33,63,64,65]. More advanced IET closures which enforce thermodynamic consistency through free parameters or resemble the VMHNC approximation by featuring an optimized correspondence rule have also been considered [66,67,68]. However, they have received comparatively much less attention than the elementary HNC and PY closures. The objective of this section is to demonstrate that the adoption of advanced IET approximations for the calculation of the static properties is an essential ingredient for reliable predictions of the MCT glass transition line. In what follows, the HNC approximation will be compared to the IEMHNC approach. The PY approximation will not be discussed owing to the long range interaction potential of interest, while the VMHNC approach will not be addressed here owing to its similar accuracy to the IEMHNC approach [58].

The discussion will center around stable rather than supercooled fluids. The main reason is that it has proven to be extremely challenging to simulate supercooled liquids due to the necessity of preventing crystallization (especially for one-component systems) [69,70,71,72,73] as well as due to the high computational cost necessary to obtain an equilibrated configuration and to effectively sample the phase space [74,75,76,77], while it is relatively cheap from a computational point of view to perform computer simulations of the stable fluid state in order to quantify the accuracy of the different IET approximations. The secondary reason is that, formally, the region of validity of the IEMHNC approach is the stable fluid region. Thus, any attempt to compare the HNC and IEMHNC approximations outside this region involves extrapolations which inevitably cast a doubt over the result of the comparison. However, as will shall deduce later, the IEMHNC approach can indeed be extrapolated deep into the supercooled liquid regime without losing its accuracy.

**Figure 1:**
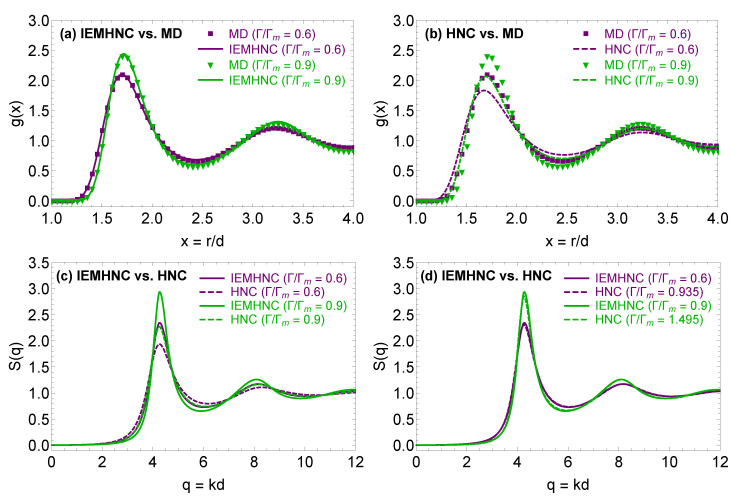
Static structural properties from the HNC and IEMHNC approaches in the stable YOCP fluid region for κ=1 with $\Gamma_{m}$ denoting the coupling parameter obtained with the analytical parameterization of the YOCP melting line given by Equation (6). Panel (**a**) compares the radial distribution function g(r) as obtained from the IEMHNC approach (solid lines) and molecular dynamics (MD) simulations (discrete points) at $\Gamma /\Gamma_{m}=\left\{0.6,0.9\right\}$. The MD simulations were performed with the LAMMPS package [78] using 8000 particles in the canonical NVT ensemble. Panel (**b**) compares the radial distribution function g(r) as obtained from the HNC approach (solid lines) and MD simulations (discrete points) at $\Gamma /\Gamma_{m}=\left\{0.6,0.9\right\}$. Panel (**c**) compares the static structure factor S(q) as obtained from the IEMHNC approach (solid lines) and the HNC approach (dashed lines), both applied at the same YOCP state points with $\Gamma /\Gamma_{m}=\left\{0.6,0.9\right\}$. Panel (**d**) compares the static structure factor S(q) stemming from the IEMHNC approach (solid lines) and the HNC approach (dashed lines), applied at the different state points, namely the IEMHNC at $\Gamma /\Gamma_{m}=\left\{0.6,0.9\right\}$ and the HNC at $\Gamma /\Gamma_{m}=\left\{0.935,1.495\right\}$.

The comparison is illustrated in Figure 1. Panels (a) and (b) feature a radial distribution function comparison between the results of the HNC, IEMHNC approximations and the results of molecular dynamics simulations. It is evident that the IEMHNC approach outperforms the HNC approximation leading to radial distribution functions that are nearly indistinguishable from the results of computer simulations within the first and second coordination cells [52]. In addition, the IEMHNC approach maintains the same level of accuracy throughout the stable fluid region [58]. On the contrary, the HNC approximation exhibits strong deviations from the MD results within the first coordination cell. More important, it becomes more and more problematic upon approaching the melting point [52,58]. The latter observation is a direct manifestation of the fact that the bridge function contribution to the static structural properties gradually becomes more prominent as the liquid–solid phase transition is approached. Finally, it is reasonable to assume that this trend continues also beyond the melting point, in the supercooled portion of the phase diagram, which implies that the HNC approximation leads to grossly inaccurate estimates for state points close to the MCT glass transition.

Panels (c) and (d) of Figure 1 feature a static structure factor comparison between the results of the HNC and IEMHNC approximations. These panels shed light on another property of the HNC approximation, which is here observed for the stable fluid region but also holds beyond the melting point (as we shall deduce in what follows); namely, the fact that the HNC approximation produces quantitatively correct structural properties but for state points of much stronger coupling than that of the actual state point. In particular, in panel (c), the IEMHNC and HNC approaches are compared for the same state points and, as anticipated, lead to different results. Given the high accuracy of the IEMHNC approach, the IEMHNC results can be considered as nearly exact which confirms that the HNC approximation introduces noticeable distortions in the static properties. On the other hand, panel (d) demonstrates that the two IET approximations result to nearly identical structural properties, if the HNC approximation is applied at $\Gamma =\Gamma_{\mathrm{HNC}}$ and the IEMHNC approach at $\Gamma =\Gamma_{\mathrm{IEMHNC}}$ with $\Gamma_{\mathrm{HNC}}\gg \Gamma_{\mathrm{IEMHNC}}$. In order to rationalize this result, it is convenient to rewrite the IET diagrammatic closure, Equation (8), as $g\left(r\right)=\exp\left[-\beta u^{*}\left(r\right)+h\left(r\right)-c\left(r\right)\right]$, with $\beta u^{*}\left(r\right)=\beta u\left(r\right)-B\left(r\right)$. This leads to the physical interpretation of the bridge function B(r) as an additional repulsion which is superimposed on the interaction potential u(r), given that it is always negative except for a series of small positive peaks in its long-range behavior [79,80,81,82]. Thus, it becomes apparent that the HNC approximation, which assumes that B(r)=0, would produce quantitatively accurate results, only when the state point is adjusted in such a way that $u^{*}\left(r\right)\approx u\left(r\right)$. For the specific case of the YOCP, this amounts to say that the HNC approximation can be expected to reproduce the structural properties of the state point (Γ,κ), only if it is applied to a different state point $\left(\Gamma_{\mathrm{HNC}}\gg \Gamma ,\kappa \right)$, as demonstrated in panel (d).

At this point, it is worth analyzing the consequences of the results discussed above on the MCT glass transition calculations. The MCT equation for the form factor, Equation (4), does not explicitly include any information about the interaction potential whose effect appears only indirectly via the static properties in the non-linear kernel stemming from the memory function. Therefore, it can be expected that the HNC approximation, which produces nearly exact static properties if the state point is artificially re-scaled towards the stronger coupling region, produces unreliable estimates for the location of the MCT glass transition line but accurate predictions for the shape of the critical MCT form factors. Therefore, for a precise determination of the MCT glass transition line, it is necessary to adopt more advanced IET closures. Here we consider two such closures, the IEMHNC approach and the VMHNC approximation. The reason behind considering both advanced closures is that none of them has been extensively tested in the supercooled regime, so it is important to confirm that they produce consistent results for a phase diagram region that falls outside their normal range of applicability.

### 3.2. Numerical Implementation

The following four-step procedure was devised for the efficient localization of the YOCP MCT glass transition line: **(i)** For a given value of the screening parameter κ, ten values of the coupling parameter Γ that belong to the YOCP supercooled region were considered according to the prescription $\Gamma_{i}=\Gamma_{i-1}+\Delta \Gamma$ with i=2,3,…10. Here $\Gamma_{1}$ and ΔΓ are κ-dependent free parameters that should be chosen in a manner that allows the exploration of a sizable portion of the YOCP supercooled region. **(ii)** For each $\left(\kappa ,\Gamma_{i}\right)$ combination, Equations (7) and (8) were solved with one of the three closures described in the Section 2.3 leading to the determination of the static structural properties of the supercooled fluid. **(iii)** For each $\left(\kappa ,\Gamma_{i}\right)$ combination, the form factor was computed from Equation (4) supplemented with the structural properties obtained from the IET equations. **(iv)** In the case of positive form factor for a state point characterized by $\Gamma =\Gamma_{i}$, the update $\Gamma_{1}\to \Gamma_{i-1}$ was employed, ΔΓ was divided by ten and the procedure was repeated until the coupling parameter of the glass transition point was determined within four digits of accuracy. In the case of zero form factors for all $\Gamma =\Gamma_{i}$ state points, the update $\Gamma_{1}\to \Gamma_{10}+\Delta \Gamma$ was employed and the procedure was restarted. In the case of positive form factors for all $\Gamma =\Gamma_{i}$ state points, the update $\Gamma_{1}\to \Gamma_{1}-11\Delta \Gamma$ was employed and the procedure was restarted. For the above procedure to prove successful, robust algorithms should be available for the solution of the IET system of Equations (7) and (8) and for the solution of the MCT form factor Equation (4). The algorithms implemented are outlined in the following paragraphs.

The IET equations were solved with a well-established algorithm [52,54,58] that is based on Picard iterations in Fourier space combined (when necessary) with mixing and long-range decomposition techniques to facilitate convergence. The Fourier transforms are computed on a discretized domain extending up to $R_{\max}=80d$ with a real space resolution $\Delta r=10^{-3}d$ and a reciprocal space resolution $\Delta k_{\mathrm{IET}}=\pi /R_{\max}=0.039/d$. The convergence criterion for the Picard iterations reads as $|\gamma_{m}\left(k\right)-\gamma_{m-1}\left(k\right)|<10^{-5}$∀k where γ(k) is the Fourier transform of the indirect correlation function γ(r)=h(r)−c(r). When the IET equations are solved within the VMHNC closure, the effective packing fraction η which appears in the VMHNC bridge function is found with a dedicated iteration cycle that is terminated when the convergence criterion $|\eta_{m}-\eta_{m-1}|/\eta_{m}<10^{-5}$ is satisfied. A more detailed description of the algorithm employed in the solution of the IET equations can be found in Ref. [58].

The MCT equation for the form factor was solved over a discretized wavenumber domain with resolution Δk=0.1/d containing 400 equally-spaced points distributed between Δk and $k_{m}=40/d$. Taking advantage of the fact that the long-time limit of the form-factor obeys the maximum property [33], Equation (4) was solved with the iterative scheme $f_{m}\left(k_{i}\right)/\left[1-f_{m}\left(k_{i}\right)\right]=M\left[f_{m-1}\left(k\right)\right]$ starting from the uniform initial guess f(k)=1.0. Here $k_{i}=i\Delta k$, $k=\{k_{1},k_{2},\ldots ,k_{M}\}$ and M[f] is a short-hand notation for the non-linear term which appears in the right hand-side of Equation (4). Concerning this non-linear term, it was first rewritten in a form more suitable for numerical integration by applying the bipolar convolution theorem [12,17]

(9) $$M\left[f\right]=\frac{nS(k)}{16\pi^{3}}\int_{0}^{\infty }dy\int_{\left|y-k\right|}^{y+k}dz\left[\left(k^{2}+y^{2}-z^{2}\right)c\left(y\right)+\left(k^{2}+z^{2}-y^{2}\right)c\left(z\right)\right]yzS\left(y\right)S\left(z\right)f\left(x\right)f\left(z\right),$$

and then it was evaluated with the adaptive Gauss-Kronrod quadrature rule as implemented in the GNU Scientific Library [83]. The discretization employed in the solution of the MCT equation has two straightforward consequences. First, the fact that $\Delta k>\Delta k_{\mathrm{IET}}$ suggests that it is necessary to interpolate the static properties obtained from IET when they are employed in the solution of the MCT equations. This, however, should not impact the final result of the MCT calculations because there is no extrapolation involved and because such results have proven to be independent from the grid resolution if Δk≤0.2/d (see Section 3.3). Second, the integrand values within the long wavelength limit (k=0) and within the short wavelength limit (k→∞) are inaccessible, since both these limits fall outside of the grid employed in the solution of the MCT equations. This implies that in Equation (9) the integration range of *y* must be shrunk from [0,∞) to $\left[\Delta k,k_{m}\right]$ and the integration range of *z* must be modified from [|y−k|,y+k] into $[\max\{\Delta k,|y-k|\},\min\left\{k_{m},y+k\right\}]$. In what follows, we shall demonstrate that the present choices for Δk and for $k_{m}$ ensure that this distorsion of the integration domain has a negligible effect on the form factors resulting from the MCT equations.

### 3.3. Benchmarking and Convergence Study

Before proceeding with the presentation of the results stemming from the systematic solution of the MCT equations over the supercooled YOCP phase diagram, it is important to discuss how the present algorithm was benchmarked against results available in the literature.

**Figure 2:**
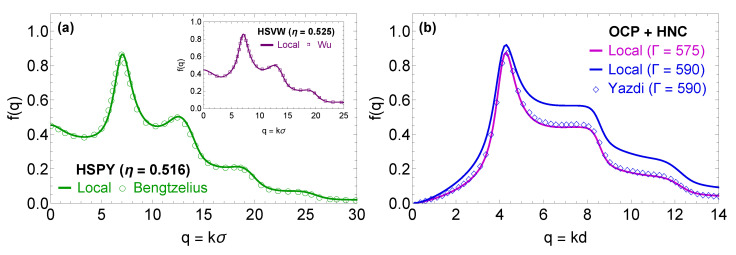
Results from the benchmarking of the algorithm employed to solve the MCT equation for the form factor, Equation (4). Panel (**a**) compares the form factor at the glass transition point for the HSPY system as obtained from the present numerical implementation (solid line) and from the results reported by Bengtzelius in Figure 2 of Ref. [17] (discrete circles). The inset compares the form factor at the glass transition point for the HSVW system as obtained from the present numerical implementation (solid line) and from the results reported by Wu and Cao in Figure 1 of Ref. [63] (discrete squares). The HS calculations were performed with $k_{m}=50/\sigma$ and Δk=0.1/σ where σ denotes the HS diameter. Panel (**b**) compares the form factor at the glass transition point for the OCP system as obtained from the present numerical implementation (solid line) and from the results reported by Yazdi and collaborators in Figure 2 of Ref. [29] (discrete diamonds). Note that for the present implementation we report the form factors at two values of the coupling parameter: Γ=575 (magenta) which is the present glass transition point prediction and Γ=590 (blue) which is the Yazdi glass transition point prediction.

The MCT benchmarking exercise initially focused on hard-sphere (HS) systems, since the glass transition of HS systems has been the focal point of numerous investigations over the years [15,16,17,33] making it relatively easy to find reference values for the critical form factor and the glass transition point. In addition, it is possible to employ analytical expressions for the static structural properties of the HS systems, which allows to test the isolated performance of the MCT solver without worrying about the performance of the IET solver. Regarding the static properties of such systems, they have either been obtained within the Percus–Yevick approximation by employing the Wertheim–Thiele analytical solution [84,85] or expressed through the accurate semi-empirical parameterization proposed by Verlet and Weis [86]. In what follows, the former case will be coined as the Hard-Sphere Percus–Yevick (HSPY) system (this is the same system employed for the definition of the bridge function in the VMHNC approximation), while the latter case will be addressed as the Hard-Sphere Verlet-Weis (HSVW) system. Panel (a) in Figure 2 illustrates how the present numerical solutions for the critical form factor of the HSPY and HSVW systems compare against results available in the literature. It is evident that the present numerical implementation is able to correctly reproduce both the glass transition points (which occur at η=0.516 for the HSPY system and η=0.525 for the HSVW system) and the corresponding critical form factors.

The MCT benchmarking exercise was also extended to YOCP systems, where the investigation of Yazdi and collaborators [29] provided the only literature results that can be employed as a reference. In panel (b) of Figure 2, the present solution of the MCT form factor equation in the OCP limit is compared with the corresponding OCP solution (as reported in Figure 2 of Ref. [29]). In both cases, the static properties of the OCP were computed by closing the IET system of equations with the HNC approximation. Two problems can immediately be noticed: the predictions for the glass transition point do not match (the present implementation predicts a glass transition at Γ=575, while the reference implementation predicts a glass transition at Γ=590) and the two implementations generate different form factors for the same coupling parameter. Unfortunately, Ref. [29] contains very little information regarding the algorithms and the parameters used to solve the MCT form factor equation. Hence, in order to rule out the presence of numerical errors in the present solution, we had to resort to a systematic convergence study on the two free numerical parameters that emerge in the solution of Equation (4) with the memory kernel expressed via Equation (9), namely the wavenumber resolution Δk and the cutoff wavenumber $k_{m}$.

**Figure 3:**
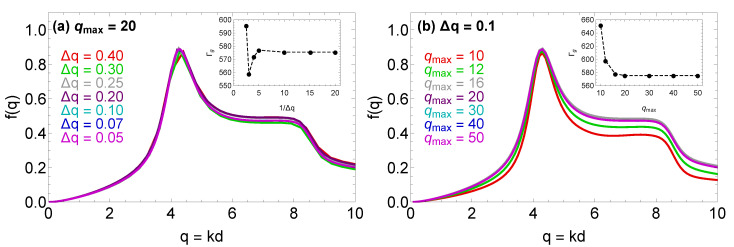
Results from the convergence study conducted in order to confirm the accuracy of the present numerical implementation of MCT for Yukawa systems. The results refer to the OCP limit (κ=0) with the structural properties computed with the HNC approximation. Panel (**a**) reports the form factor at the glass transition point for a fixed value of the cutoff wavenumber $q_{m}=k_{m}d=20$ and for seven color-coded values of the grid resolution, namely Δq=Δkd={0.05,0.07,0.10,0.20,0.25,0.30,0.40}. The inset illustrates how the coupling parameter at the glass transition point, $\Gamma_{g}$, converges towards $\Gamma_{g}=575$ upon increasing the grid resolution. Panel (**b**) reports the form factor at the glass transition point for a fixed value of the grid resolution Δq=0.1 and seven color-coded values of the cutoff wavenumber, i.e., $q_{m}=\left\{10,\;12,\;16,\;20,\;30,\;40,\;50\right\}$. The inset illustrates how the coupling parameter at the glass transition point, $\Gamma_{g}$, converges towards $\Gamma_{g}=575$ upon increasing the cutoff wavenumber.

In the convergence study, the wavenumber resolution was varied between Δk=0.05/d and Δk=0.4/d, while the cutoff wavenumber was varied between $k_{m}=10/d$ and $k_{m}=50/d$. The results of this investigation are reported in Figure 3 and reveal that a wavenumber resolution Δk≤0.2/d and a cutoff wavenumber $k_{m}\geq 20/d$ are sufficient to obtain a form factor and glass transition point $\Gamma_{g}$ which are independent from the parameters employed in the numerical implementation. The results in Figure 3 refer to the OCP limit (κ=0) and to structural input from the HNC approximation, but similar conclusions were obtained also for selected higher values of κ and structural input from the IEMHNC or VMHNC approximations.

To summarize, this section demonstrated that the present numerical implementation of the MCT equations is able to reproduce results available in the literature for HS systems. Regarding YOCP systems, a small mismatch was observed between the present results and the literature results. However, while it was not possible to pin-point the origin of this mismatch due to insufficient information, the results of a thorough convergence study ensured that the present MCT calculations are accurate also for Yukawa systems.

## 4. Numerical Results

### 4.1. The MCT Glass Transition Line

The glass transition line of the YOCP was computed for fifteen screening parameters which belong to the part of the YOCP phase diagram that is most relevant to experimental realizations of Yukawa systems and for which molecular dynamics based calculations of the liquid–solid phase transition are available [24,42], κ={0,0.2,0.4,0.6,0.8,1.0,1.2,1.4,2.0,2.6,3.0,3.6,4.0,4.6,5.0}. For each value of κ, the MCT glass transition point was localized with the procedure described in Section 3.2. Three sets of MCT calculations have been performed in order to determine the glass transition line featuring different static properties for the supercooled YOCP fluid, i.e., those computed with the HNC approximation (MCT-H calculations), the IEMHNC approach (MCT-I calculations) and the VMHNC approximation (MCT-V calculations).

The phase diagram coordinates of the glass transition line obtained with the MCT-H, MCT-I and MCT-V calculations are reported in Table 1. It is apparent that the MCT-H estimate of the glass transition line is drastically different from the MCT-I and MCT-V estimates, whereas the MCT-I and MCT-V estimates lie relatively close to each other. In particular, considering $\Gamma_{g}^{\mathrm{MCT}-I}\left(\kappa \right)$ as a reference curve, the MCT-V prediction falls within 5% of the MCT-I result, $\Gamma_{g}^{\mathrm{MCT}-V}\left(\kappa \right)=\left(1\pm 0.05\right)\Gamma_{g}^{\mathrm{MCT}-I}\left(\kappa \right)$, while the MCT-H estimate is approximately given by $\Gamma_{g}^{\mathrm{MCT}-H}\left(\kappa \right)\approx 2\Gamma_{g}^{\mathrm{MCT}-I}\left(\kappa \right)$ for any value of the screening parameter.

The observation that $\Gamma_{g}^{\mathrm{MCT}-V}\left(\kappa \right)\approx \Gamma_{g}^{\mathrm{MCT}-I}\left(\kappa \right)$ suggests that the IEMHNC approximation and the VMHNC approach maintain the consistency and accuracy which characterizes them for stable YOCP liquids also for supercooled YOCP liquids. In addition, it indirectly suggests that the defacto extrapolations of the IEMHNC and VMHNC bridge functions deep into the supercooled regime are rather safe. As a consequence, both the $\Gamma_{g}^{\mathrm{MCT}-V}\left(\kappa \right)$ and $\Gamma_{g}^{\mathrm{MCT}-I}\left(\kappa \right)$ estimates can be considered to be an accurate approximation to the “exact” MCT glass transition line which would have been obtained if the MCT equations were supplied with an “exact” structural input extracted from computer simulations. On the other hand, the observation that $\Gamma_{g}^{\mathrm{MCT}-H}\left(\kappa \right)\approx 2\Gamma_{g}^{\mathrm{MCT}-I}\left(\kappa \right)$ confirms that adopting the HNC approximation would lead to inaccurate estimates for the MCT glass transition line, see the detailed discussion in Section 3.1. In fact, assuming that the MCT-I or MCT-V calculations are able to predict the “exact” MCT glass transition line within a few percent, then the MCT-H calculations lead to an overestimation of the MCT glass transition line of roughly a factor two, regardless of the value of the screening parameter. This justifies our revisiting of the YOCP glass transition results earlier reported by Yazdi and collaborators [29], since the latter were exclusively based on MCT-H calculations.

Perhaps, the most significant result of Ref. [29] was the observation that, for YOCP systems, the glass transition line is almost parallel to the melting line. As a consequence, it can be well approximated by the following analytical semi-empirical formula [29]

(10) $$\Gamma_{g}\left(\kappa \right)=\Gamma_{g}^{\mathrm{OCP}}e^{\alpha \kappa }{\left[1+\alpha \kappa +\frac{{\left(\alpha \kappa \right)}^{2}}{2}\right]}^{-1},$$

where $\Gamma_{g}^{\mathrm{OCP}}$ is the glass transition point in the OCP limit. It is important to test the validity of the scaling given in Equation (10) against the accurate glass transition predictions obtained from the MCT-I and the MCT-V calculations not only because it provides a simple convenient parameterization but also because its validity is a strong indication that the glass transition line constitutes an isomorphic curve, compare Equations (10) and (5). As we shall see in the following sections, the isomorph invariance of the MCT glass transition line implies the validity of numerous state-independent symmetries that can be exploited in different ways.

**Figure 4:**
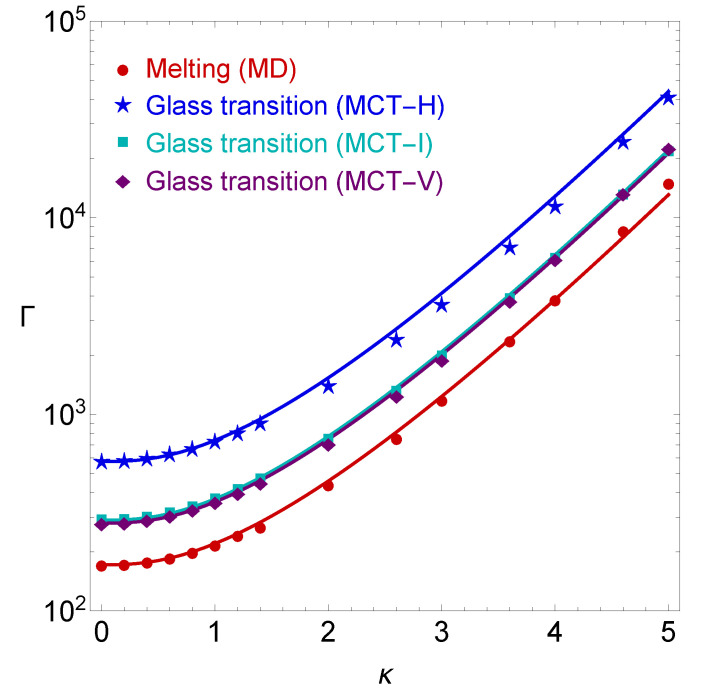
YOCP phase diagram featuring the liquid–solid phase transition line determined from the MD simulations of Ref. [24] (circles), the glass transition line determined from the MCT-H calculations (stars), the glass transition line determined from the MCT-I calculations (squares), the glass transition line determined from the MCT-V calculations (diamonds). The solid curves represent the prediction of the semi-empirical expression for the melting line of Equation (6) with $\Gamma_{m}^{\mathrm{OCP}}=171.8$ (red) and the prediction of the semi-empirical expression for the glass transition line of Equation (10) with $\Gamma_{g}^{\mathrm{OCP}}=575.5$ (blue), with $\Gamma_{g}^{\mathrm{OCP}}=289.8$ (cyan) and with $\Gamma_{g}^{\mathrm{OCP}}=279.7$ (purple).

In Figure 4, the analytical scaling of Equation (10) is compared against the predictions of the three MCT glass transition calculations. At this point, it should be noted that during the comparison with a type of MCT calculation, the $\Gamma_{g}^{\mathrm{OCP}}$ pre-factor which appears in Equation (10) is adapted to match to the corresponding value that is given in the first line of Table 1. This means that $\Gamma_{g}^{\mathrm{OCP}}=575.5$ for MCT-H, $\Gamma_{g}^{\mathrm{OCP}}=289.8$ for MCT-I and $\Gamma_{g}^{\mathrm{OCP}}=279.7$ for MCT-V. It is apparent that the semi-empirical expression performs well for the MCT-H calculations (as it was already observed in Ref. [29]) and that it performs even better for the MCT-I calculations and the MCT-V calculations. To be more quantitative, for the set of screening parameters considered in this work (κ≤5.0), the relative deviations between the analytical scaling and $\Gamma_{g}^{\mathrm{MCT}-H}\left(\kappa \right)$ can become as large as 15%, while the relative deviations between the analytical scaling and $\Gamma_{g}^{\mathrm{MCT}-I}\left(\kappa \right)$ or $\Gamma_{g}^{\mathrm{MCT}-V}\left(\kappa \right)$ never exceed 5%.

The high accuracy of analytical semi-empirical expression for the MCT glass transition and the equivalence of Equation (10) to Equation (5) with Λ=1, suggest that the glass transition line can be considered to be an isomorphic curve. This observation also rationalizes why the analytical scaling agrees better with the MCT-I calculations which feature an explicitly isomorph-invariant bridge function [52] and with the MCT-V calculations which feature an implicitly isomorph-invariant bridge function [58] rather than with the MCT-H calculations which neglect the bridge function altogether. It should be mentioned that a modified version of Equation (10) was also tested, in which the analytical scaling was made fully equivalent to the expression for the YOCP isomorphs by replacing ακ with Λακ and then by determining Λ via least-square fitting. The results of such modified scaling will not be analyzed further, because for all three calculations the parameter Λ was found to be very close to unity, suggesting that the choice Λ=1 is already nearly optimal, and that the modified scaling possesses essentially the same accuracy of the standard scaling of Equation (10).

The above results indicate that the MCT glass transition line of YOCP systems constitutes an isomorphic curve. Given that isomorphs are phase diagram lines of constant reduced excess entropy, this suggestion can be supported by computing the reduced excess entropies of the YOCP state points that lie along the glass transition line, $s_{\mathrm{ex}}\left(\kappa ,\Gamma_{g}\right)$, and then by checking to which extent they satisfy the condition $s_{\mathrm{ex}}\left(\kappa ,\Gamma_{g}\right)=\mathrm{const}.$ The accurate computation of the excess entropy requires to perform computer simulations of supercooled fluids combined with free-energy calculation techniques [87], but the correct implementation and execution of such simulations falls outside the scope of the present work. Hence, here we computed $s_{\mathrm{ex}}\left(\kappa ,\Gamma_{g}\right)$ by utilizing two YOCP equations of state available in the literature which provide the reduced excess internal energy, $u_{\mathrm{ex}}\left(\kappa ,\Gamma \right)$, from which the reduced excess entropy can be straightforwardly computed by thermodynamic integration from $s_{\mathrm{ex}}\left(\kappa ,\Gamma \right)=u_{\mathrm{ex}}\left(\kappa ,\Gamma \right)-\int_{0}^{\Gamma }u_{\mathrm{ex}}\left(\kappa ,\Gamma^{\prime }\right)/\Gamma^{\prime }$. Note that reduced excess internal energies are expressed in units of $Nk_{b}T$ and reduced excess entropies are expressed in units of $Nk_{b}$.

The equation of state constructed by Hamaguchi and coworkers fits well computer simulation results in the entire stable fluid YOCP phase diagram and expresses the reduced excess internal energy as $u_{\mathrm{ex}}^{H}\left(\kappa ,\Gamma \right)=a\left(\kappa \right)\Gamma +b\left(\kappa \right)\Gamma^{1/3}+c\left(\kappa \right)+d\left(\kappa \right)\Gamma^{-1/3}$ [24,42]. The equation of state proposed by Rosenfeld and Tarazona, within the framework of an asymptotically-high density expansion for purely repulsive potentials, fits well computer simulation results in the strongly coupled stable liquid portion of the YOCP phase diagram and leads to the reduced excess internal energy expression $u_{\mathrm{ex}}^{\mathrm{RT}}\left(\kappa ,\Gamma \right)=M\left(\kappa \right)\Gamma +3.0{\left[\Gamma /\Gamma_{m}\left(\kappa \right)\right]}^{2/5}$ [88,89]. The κ-dependent parameters a(κ),b(κ),c(κ),d(κ) and M(κ) are given in the respective references, while the coupling parameter at the melting point, $\Gamma_{m}\left(\kappa \right)$, can be conveniently expressed via Equation (6) [54]. It should be emphasized that both equations of state are strictly valid only for the stable fluid phase of the YOCP. In order to confirm that they can be safely extrapolated to the supercooled regime, the reduced excess internal energies $u_{\mathrm{ex}}^{H}\left(\kappa ,\Gamma \right)$ and $u_{\mathrm{ex}}^{\mathrm{RT}}\left(\kappa ,\Gamma \right)$ were compared against those computed from the IET structural properties, $u_{\mathrm{ex}}\left(\kappa ,\Gamma \right)=\left(3/2\right)\int_{0}^{\infty }x^{2}\beta u\left(x\right)g\left(x\right)dx$, along the glass transition line that stems from the MCT-I and MCT-V calculations. The comparison showed that both equations of state could predict the reduced excess internal energy within 2% along the MCT-I glass transition line and within 1% along the MCT-V glass transition line leading to the conclusion that both can be employed to obtain accurate estimates for thermodynamic properties of supercooled Yukawa fluids.

The reduced excess entropies along the MCT-I and MCT-V glass transition lines are reported in Table 2. The MCT-H calculations are not included due to their poor glass transition line prediction. There are deviations in the entropy predictions of the equations of state. The Rosenfeld–Tarazona expression predicts that both glass transition lines satisfy $s_{\mathrm{ex}}\left(\kappa ,\Gamma_{g}\right)=-5.5$ within 3%, whereas the Hamaguchi expression predicts reduced excess entropies that are not constant along the glass transition lines (see the relatively large deviations reported in the last two rows of Table 2) and change noticeably between the MCT-I and MCT-V glass transition lines. At this point, one should recall that the Hamaguchi equation of state was constructed on an empirical basis by observing the functional dependencies within the stable fluid range, while the Rosenfeld–Tarazona equation of state was derived on the basis of an asymptotically-high coupling parameter expansion that ignores the liquid–solid phase transition. Thus, the Rosenfeld–Tarazona equation of state should be more accurate for supercooled YOCP liquids. Overall, from the results of Table 2, it can be concluded that the MCT glass transition line constitutes an isentropic curve with $s_{\mathrm{ex}}=-5.5$ (within 3%) reduced excess entropy.

### 4.2. The MCT form Factors

**Figure 5:**
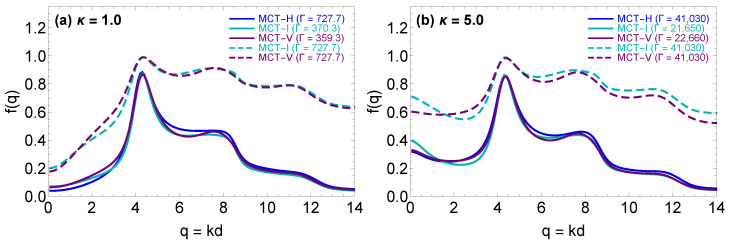
Comparison between the critical YOCP form factors stemming from the MCT-H (blue), MCT-I (cyan) and MCT-V (purple) calculations for two values of the screening parameter, κ=1.0 in panel (**a**), κ=5.0 in panel (**b**). Each panel also reports the form factors obtained from the MCT-I and MCT-V calculations at the glass transition state point predicted by the MCT-H calculations (dashed lines).

The critical form factors resulting from the MCT-H, MCT-I and MCT-V calculations are compared in Figure 5 for two screening parameters. Important observations from Figure 5 are that the MCT-H, MCT-I and MCT-V calculations lead to similar form factors at their respective glass transition points (compare the solid curves) as well as that there are enormous form factor differences between MCT-H and MCT-I or MCT-V calculations at the same state point (compare the dashed curves to the blue solid curve). These observations are in line with the discussions of Section 2.3 and Section 3.1. They are both manifestations of the fact that the MCT non-linear kernel on the right hand side of Equation (4) requires only the static structure factor as external input and does not contain any explicit dependence on the interaction potential. The form-factor similarity at the glass transition point can be explained by the fact that the IEMHNC, VMHNC and HNC approximations can all be expected to produce similar structure factors at their respective glass transition points; the IEMHNC and VMHNC approximations due to their high accuracy (see Section 2.3) and the HNC for the reasons discussed in Section 3.1. On the other hand, the large discrepancies observed between the MCT-H calculations and the other two sets of calculations at the same state point are caused by the poor performance of the HNC approximation in the dense fluid region which was also discussed in Section 3.1.

**Figure 6:**
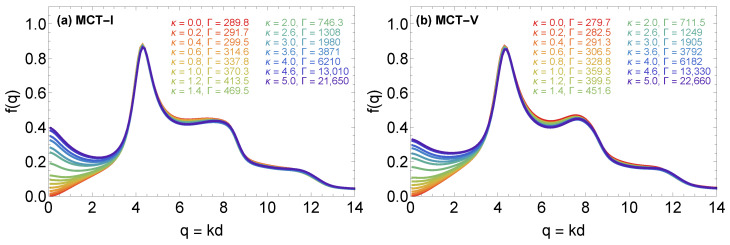
Critical YOCP form factors along the MCT-I (panel **a**) and the MCT-V glass transition line (panel **b**). Each panel reports the results for fifteen YOCP state points with screening parameters κ={0.0,0.2,0.6,0.8,1.0,1.2,1.6,2.0,2.6,3.0,3.6,4.0,4.6,5.0} (color-coded).

**Figure 7:**
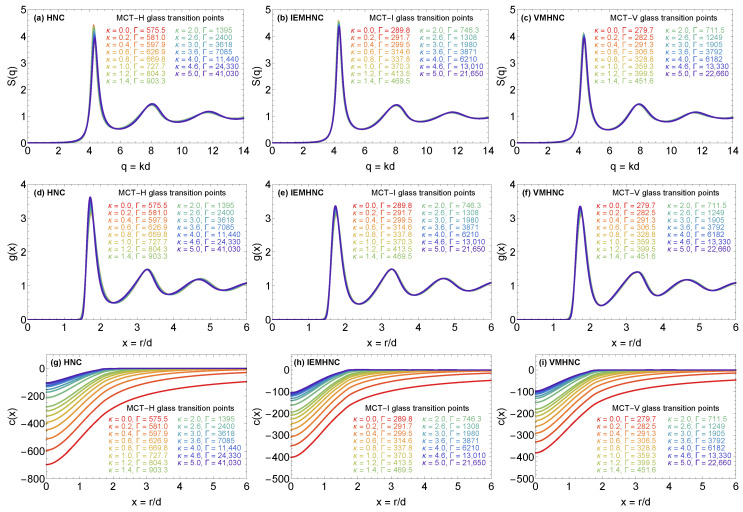
YOCP static properties obtained by solving the IET Equations (7), (8) with the HNC closure along the MCT-H glass transition line (panels **a**,**d**,**g**), with the IEMHNC closure along the MCT-I glass transition line (panels **b**,**e**,**h**) and with the VMHNC closure along the MCT-V glass transition line (panels **c**,**f**,**i**). The static structure factor is presented in panels (**a**–**c**), the radial distribution function in panels (**d**–**f**) and the direct correlation function in panels (**g**–**i**). Each panel reports the results for 15 state points with screening parameters κ={0.0,0.2,0.6,0.8,1.0,1.2,1.6,2.0,2.6,3.0,3.6,4.0,4.6,5.0} (color-coded).

Figure 6 illustrates how the critical YOCP form factor changes with the screening parameter according to MCT-I and MCT-V calculations; similar results (not shown here) were also obtained from MCT-H calculations. Regardless of the IET approximation employed to compute the static structural properties, it is apparent that the critical form factor is characterized by a main peak whose magnitude and position are practically independent of the screening parameter under consideration. The main peak is preceded by a rapid decay towards a state-point-dependent long wavelength limit and is followed by slowly decaying oscillations which also appear to be state-point-independent. The results of Figure 6 are rather anticipated in light of our previous discussions. Since the MCT glass transition line constitutes an isomorphic curve, then a large number of reduced unit dynamic and structural properties should be nearly invariant while traversing the glass transition line. The static structure factor is known to be an isomorph invariant quantity, thus, given the deep connection between S(k) and f(k), it can be expected that also the form factor exhibits some degree of invariance. On the other hand, the variance of f(k) near the k=0 limit is caused by the fact that the long wavelength limit of the MCT memory kernel is given by a relation of the form $\alpha S\left(0\right)=\alpha \chi_{T}$ where the pre-factor α is described by Equation (9b) of Ref. [29] and where $\chi_{T}=1+n\int h\left(r\right)d^{3}r$ is the isothermal compressibility, which is known to strongly vary along an isomorph [90] (strictly speaking, $S\left(0\right)=\chi_{T}$ does not hold for the OCP for which the correct infinite wavelength limit of the structure factor, S(0)=0, must be retrieved from the small argument expansion $S\left(q\to 0\right)={\left(3\Gamma /q^{2}+1/\chi_{T}\right)}^{-1}$ with q=kd [91]). While the isomorph variance of S(0) has a negligible effect on the overall degree of invariance of the static structure factor due to the nearly incompressible nature of supercooled YOCP liquids (see Figure 7), the isomorph variance of S(0) has a strong effect on the degree of invariance of the form factor over a sizeable wavenumber interval due to the large value of the proportionality constant α which augments the small compressibility values.

### 4.3. The MCT Vitrification Indicators

The YOCP static structure factor, radial distribution function and direct correlation function along the MCT glass transition line (MCT-H, MCT-I, MCT-V) are illustrated in Figure 7. Let us first focus on the results for the state points pertaining to the MCT-I and MCT-V glass transition lines. In accordance with the predictions of isomorph theory, the static structure factor and radial distribution function are almost invariant. On the other hand, owing to the asymptotic limit c(r→∞)=−βu(r) and due to the connection to the compressibility via $\left(1/\chi_{T}\right)=-n\int c\left(r\right)d^{3}r$, the direct correlation function is strongly variant. Albeit not directly evident from Figure 7, the radial distribution function and the static structure factor are similar to f(k) in the sense that their short-range values are also non-invariant; g(r) is variant due to the asymptotic limit g(r→0)∝exp[−βu(r)] [92], while S(k) is variant due to its connection to the compressibility $S\left(k\to 0\right)=\chi_{T}$. However, while the short-range variance has a noticeable effect on the critical form factor, it is inconsequential for both the radial distribution function and the static structure factor since it occurs in a region where both functions are approximately zero. Finally, we point out that the invariance of the radial distribution and the static structure factor is slightly violated in the vicinity of their first maxima, but it holds to a nearly exact degree at their subsequent minima and maxima. Regarding the static properties along the MCT-H glass transition line, it can be noticed that S(k) and g(r) both show a reduced degree of invariance, in accordance to an earlier observation which reported that assuming B(r)=0 has a detrimental effect on the invariant properties along an isomorphic line [52].

The high degree of isomorph invariance which characterizes the static structure factor and the radial distribution function at the MCT glass transition line opens up the possibility of determining a group of empirical vitrification indicators. Such indicators are inspired by the successful application of freezing indicators which allow to locate the liquid–solid phase transition line simply by monitoring some characteristic features of the liquid state S(k) and g(r). Empirical vitrification indicators would serve as phenomenological criteria for the localization of the glass transition line based solely on the structural properties of the supercooled liquid. In fact, it was observed that two commonly used freezing indicators, namely the magnitude of the first peak of the static structure factor $S_{\max}$ employed in the Hansen–Verlet freezing rule [93] and the amplitude ratio of the first nonzero minimum to the first maximum of the radial distribution function $g_{R}$ employed in the Raveché–Mountain–Streett freezing rule [94] perform reasonably well also for the prediction of the glass transition line, albeit at different values. In particular, it was revealed that the MCT-I glass transition line is characterized by $S_{\max}=4.49\pm 0.15$ and by $g_{R}=0.14\pm 0.01$, while the MCT-V glass transition line is characterized by $S_{\max}=4.00\pm 0.08$ and by $g_{R}=0.13\pm 0.01$.

Given the fact that most of the non-invariant features of S(k) and g(r) are concentrated around their first peak and taking into account that an effective vitrification indicator should remain as constant as possible along the glass transition line, it should be possible to identify even better vitrification indicators by generating simple curve metrics that do not involve the magnitude of the first peak of these quantities. For this reason, two additional prospective vitrification indicators were considered which refer to the amplitude ratio of the first nonzero minimum to the second maximum of the radial distribution function, $g_{R2}$, and the amplitude ratio of the first nonzero minimum to the second maximum of the static structure factor, $S_{R2}$. The values of these prospective vitrification indicators along the glass transition lines stemming from the MCT-I and the MCT-V calculations are reported in Table 3.

**Figure 8:**
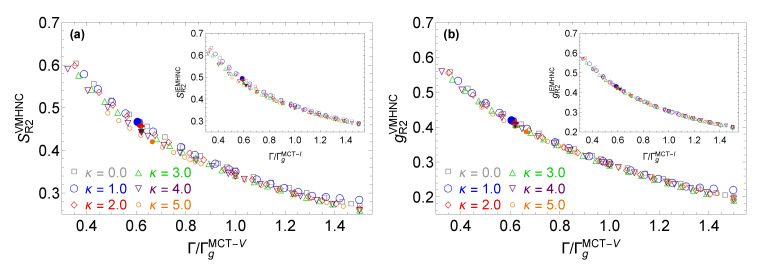
The variations of the prospective vitrification indicators $S_{R2}$ (panel **a**) and $g_{R2}$ (panel **b**) for different screening parameters as a function of the coupling parameter normalized by its MCT glass transition value. Results for stable liquids ($\Gamma /\Gamma_{g}\left(\kappa \right)≲0.6$), supercooled liquids prior to the glass transition $\left(0.6≲\Gamma /\Gamma_{g}\left(\kappa \right)<1.0\right)$ and supercooled liquids post the glass transition $\left(\Gamma /\Gamma_{g}\left(\kappa \right)>1.0\right)$. For each indicator, the superscript denotes the IET approximation employed, i.e., the VMHNC approach or IEMHNC approach. The panels report the $S_{R2}$ or $g_{R2}$ values for six screening parameters κ={0,1,2,3,4,5} and two MCT glass transition lines, namely those stemming from MCT-V calculations where $\Gamma_{g}\left(\kappa \right)\equiv \Gamma_{g}^{\mathrm{MCT}-V}\left(\kappa \right)$ (main plot) and from MCT-I calculations where $\Gamma_{g}\left(\kappa \right)\equiv \Gamma_{g}^{\mathrm{MCT}-I}\left(\kappa \right)$ (inset). The numerical values of $\Gamma_{g}^{\mathrm{MCT}-V}\left(\kappa \right)$ and $\Gamma_{g}^{\mathrm{MCT}-I}\left(\kappa \right)$ have been reported in Table 1. The full symbols represent the values of $S_{R2}$ and $g_{R2}$ at the YOCP melting point predicted by computer simulations [24].

Both prospective vitrification indicators remain almost constant along the MCT glass transition line, with minor deviations from their average value which do not exceed 1% for $g_{R2}$ and 3% for $S_{R2}$. These vitrification indicators possess another desirable property, since they exhibit respectable variations within the supercooled regime prior to and post the glass transition line (see Figure 8). This characteristic highlights their potential practical use for the localization of the MCT glass transition point. Overall, the indicators $g_{R2}$ and $S_{R2}$ perform better than $S_{\max}$ and $g_{R}$, since they exhibit slightly smaller variations along the MCT glass transition line and result to more consistent predictions between the two IET approaches employed in the computation of the MCT glass transition line. In this regard, the VMHNC approximation and the IEMHNC approach produce really close but not identical values for $g_{R2}$ and $S_{R2}$ at the glass transition point. We argue that the results obtained with the VMHNC approximation should be preferred over the ones obtained with the IEMHNC approach, since the former approximation is known to produce more accurate predictions for the second coordination cell [58]. Combining the above, it can be concluded that the MCT glass transition point is characterized by $g_{R2}=0.30$ or equivalently by $S_{R2}=0.35$ and that these two conditions can be employed to obtain an accurate guess for the MCT glass transition line of the YOCP without having to solve the MCT equation. On a side note, it is worth pointing out that the vitrification indicator $g_{R2}$ could also be employed as a freezing indicator, since the state points along the YOCP melting line obtained via computer simulations are all characterized by $g_{R2}=0.4\pm 0.01$ (see panel b in Figure 8).

## 5. Discussion and Future Work

### 5.1. Summary of the Results

In the present work, the glass transition line of Yukawa one-component plasmas was computed by combining the mode coupling theory of the glass transition with highly accurate structural input obtained from two advanced closures to the integral equation theory of liquids, namely the isomorph-based empirically modified hypernetted chain approach and the variational modified hypernetted chain approach. It was observed that both closures lead to consistent values for the YOCP glass transition line and it was concluded that the present results offer a greatly improved estimate compared to earlier estimates that are available in the literature. Besides the improvement upon existing results, the highly accurate structural input adopted in the present calculations allowed the identification of two vitrification indicators which can be employed to obtain an accurate guess for the YOCP glass transition line without necessitating a determination of the bifurcation point. The existence of vitrification indicators is important from a theoretical and practical standpoint; from a theoretical perspective, the possibility to identify reliable vitrification indicators that are solely based on the structural properties of the supercooled fluid is a direct manifestation of the fact that the glass transition line is an isomorph. From an experimental perspective, the vitrification indicators can be used to guide experiments aimed at reaching the glassy state since they can be readily estimated from the radial distribution function or the static structure factor, two quantities which are often easily measured in the course of an experiment either by direct camera observation in the case of soft matter [95] or by neutron diffraction in the case of atomic or molecular systems [96].

### 5.2. Extension to Bi-Yukawa Systems

The present results can be trivially extended to bi-Yukawa systems, which are one-component model systems relevant to the laboratory realization of complex plasmas [97,98] that are characterized by purely repulsive interactions of the form $\beta u\left(x\right)=\left(\Gamma /x\right)[\left(1-\sigma \right)e^{-\kappa x}+\sigma e^{-\mu \kappa x}]$, where σ∈[0,0.5] and μ∈[0,1] are external (non-thermodynamic) potential parameters. For such systems, it has been demonstrated that the isomorphs can be accurately parameterized by the analytical expression [54] $\Gamma \{\left(1-\sigma \right)e^{-\alpha \kappa }\left[1+\alpha \kappa +{\left(\alpha \kappa \right)}^{2}/2\right]+\sigma e^{-\alpha \mu \kappa }\left[1+\alpha \mu \kappa +{\left(\alpha \mu \kappa \right)}^{2}/2\right]\}=\mathrm{const}.$ Given that the glass transition line is nearly an isomorph, it can be expected that the bi-Yukawa glass transition line is given by $\Gamma_{g}^{\mathrm{biYOCP}}\left(\kappa ;\sigma ,\mu \right)$$=\Gamma_{g}^{\mathrm{OCP}}{\left\{\left(1-\sigma \right)e^{-\alpha \kappa }\left[1+\alpha \kappa +{\left(\alpha \kappa \right)}^{2}/2\right]+\sigma e^{-\alpha \mu \kappa }\left[1+\alpha \mu \kappa +{\left(\alpha \mu \kappa \right)}^{2}/2\right]\right\}}^{-1}$ with $\Gamma_{g}^{\mathrm{OCP}}\approx 280$. An identical expression for $\Gamma_{g}^{\mathrm{biYOCP}}\left(\kappa ;\sigma ,\mu \right)$ was earlier inferred by Yazdi and collaborators [29] who, however, overestimated $\Gamma_{g}^{\mathrm{OCP}}$ by approximately a factor of two.

### 5.3. Quasi-Universality Aspects

A brief investigation of the glass transition point for the HSPY system and for three inverse power law (IPL) systems with exponents *m* equal to 4, 9 and 12 revealed that the structural vitrification indicators $S_{R2}$ and $g_{R2}$ have a quasi-universal character, i.e., the conditions $S_{R2}=0.35$ and $g_{R2}=0.30$ produce an accurate estimate for the glass transition point regardless of the system under consideration. In the investigation of the HSPY and IPL-m glass transition point, we proceeded as follows: (**a**) The static properties of the HSPY system were computed via the Wertheim–Thiele analytical solution [84,85], while the static structure factors for the IPL-4, IPL-9, IPL-12 systems were computed with the VMHNC approach. (**b**) MCT was employed for the IPL systems leading to the glass transition points $\tilde{n}=8.103$ for IPL-4, $\tilde{n}=1.648$ for IPL-9 and $\tilde{n}=1.322$ for IPL-12 where $\tilde{n}={\left(\beta \epsilon \right)}^{3/m}n\sigma_{\mathrm{IPL}}$ is a temperature-scaled density which fully specifies the state point of an IPL-m system with interaction potential $u\left(r\right)=\epsilon {\left(\sigma_{\mathrm{IPL}}/r\right)}^{m}$. On the other hand, it was not necessary to solve the MCT for the HSPY system, since it is known that it vitrifies when the packing fraction becomes η=0.516, see Section 3.3. (**c**) The vitrification indicators were computed and it was revealed that the four systems satisfied $S_{R2}=0.35$ and $g_{R2}=0.30$ within 3% at their respective glass transition points.

In addition, the quasi-universal character of the thermodynamic vitrification indicator $s_{\mathrm{ex}}=-5.5$, obtained in Section 4.1, was tested. The reduced excess entropy of the HSPY system was computed from the Wertheim–Thiele equation of state [85] and the reduced excess entropy of the IPL systems was computed with the Rosenfeld equation of state [88]. These led to $s_{\mathrm{ex}}^{\mathrm{HSPY}}\left(\eta =0.516\right)=-5.6$, $s_{\mathrm{ex}}^{\mathrm{IPL}-4}\left(\tilde{n}=8.103\right)=-6.3$, $s_{\mathrm{ex}}^{\mathrm{IPL}-9}\left(\tilde{n}=1.648\right)=-5.3$ and $s_{\mathrm{ex}}^{\mathrm{IPL}-12}\left(\tilde{n}=1.322\right)=-4.7$. While the HSPY result for compares well with the YOCP result, the IPL results exhibit large deviations from $s_{\mathrm{ex}}=-5.5$. Such deviations are probably connected to the unjustified extrapolation of the IPL equation of state in the supercooled regime, but this can only be confirmed by an extended IPL investigation which falls beyond the scope of the present work.

### 5.4. Future Work

The traditional mode coupling theory of the glass transition has some drawbacks that stem from the assumptions that are necessary in order to derive a tractable equation for the intermediate scattering function. In fact, MCT predicts a glass transition at higher temperatures than the ones observed in experiments [22]. For the YOCP, this implies that the true glass transition point will occur at coupling parameters which are higher than the ones predicted by MCT.

A possible approach to rigorously improve the MCT predictions is to employ the so-called Generalized mode coupling theory (GMCT) of the glass transition [63,99]. Similar to MCT, GMCT is an entirely first-principle approach which allows to describe the physics of the glass transition without having to resort to computer simulations or experiments. The GMCT tries to improve upon the MCT by delaying the uncontrolled factorization of the density correlation functions, which is necessary to obtain a simplified version of the memory kernel. In doing so, the GMCT derives an infinite hierarchy of equations of motion in which the *N*-particle density correlation function depends on the (N+2)-particle density correlation function. The hierarchy is then truncated at some finite order $N=N_{\max}$ and it is assumed that the correlations for $N>N_{\max}$ can be factorized with an MCT-like approach. If $N_{\max}=2$, GMCT simply reduces to MCT.

It has been observed that the delayed factorization of the correlation functions employed in GMCT has a beneficial effect in correcting some short-comings of the MCT; most noticeably it has been proven that truncating the GMCT at $N_{\max}=4$ or at $N_{\max}=6$ brings the estimate for the HS glass transition point systematically closer to the experimental value of η=0.580 obtained with colloidal hard-spheres [63,99]. Future developments of the current results could go in the direction of employing GMCT to refine the present estimates of the YOCP glass transition line. Given that, similarly to the MCT equations, the GMCT equations do not contain any explicit dependence on the interaction potential, it can be expected that the improvements observed for HS systems would also appear for the YOCP.

## Figures and Tables

**Table 1:** YOCP phase diagram coordinates of the MCT glass transition line at fifteen values of the screening parameter, κ={0,0.2,0.4,0.6,0.8,1.0,1.2,1.4,2.0,2.6,3.0,3.6,4.0,4.6,5.0}. For each value of κ, three estimates of the coupling parameter at the MCT glass transition line, $\Gamma_{g}\left(\kappa \right)$, are reported. Each $\Gamma_{g}\left(\kappa \right)$ estimate was computed with static structure factor input from a different IET closure. Namely, $\Gamma_{g}^{\mathrm{MCT}-H}$ is obtained with the HNC approximation (MCT-H calculations), $\Gamma_{g}^{\mathrm{MCT}-I}$ with the IEMHNC approximation (MCT-I calculations) and $\Gamma_{g}^{\mathrm{MCT}-V}$ with the VMHNC approximation (MCT-V calculations).

κ	$\Gamma_{g}^{\mathrm{MCT}-H}$	$\Gamma_{g}^{\mathrm{MCT}-I}$	$\Gamma_{g}^{\mathrm{MCT}-V}$
0.0	575.5	289.8	279.7
0.2	581.0	291.7	282.5
0.4	597.9	299.5	291.3
0.6	626.9	314.6	306.4
0.8	669.8	337.8	328.8
1.0	731.1	370.3	359.3
1.2	804.3	413.5	399.5
1.4	903.3	469.5	451.6
2.0	1395	746.3	711.5
2.6	2400	1308	1249
3.0	3618	1980	1905
3.6	7085	3871	3792
4.0	11,440	6210	6182
4.6	24,430	13,010	13,330
5.0	41,030	21,650	22,660

**Table 2:** Excess entropy along at the MCT-I glass transition line (columns 3 and 4) and the MCT-V glass transition line (columns 6 and 7) as obtained from the Hamaguchi equation of state $s_{\mathrm{ex}}^{H}$, and the Rosenfeld–Tarazona equation of state, $s_{\mathrm{ex}}^{\mathrm{RT}}$. All the excess entropies are reported in units of $Nk_{b}$, i.e., these are reduced excess entropies. In the last three rows, the designation AVE denotes the average value of the reduced excess entropy along the respective glass transition line (rows 1–15), while the quantities $\epsilon_{a}$ and $\epsilon_{m}$ report the average and maximum relative deviations between the reduced excess entropies along the respective glass transition line and their average value reported in AVE.

κ	$\Gamma_{g}^{\mathrm{MCT}-I}$	$s_{\mathrm{ex}}^{H}\left(\mathrm{MCT}-I\right)$	$s_{\mathrm{ex}}^{\mathrm{RT}}\left(\mathrm{MCT}-I\right)$	$\Gamma_{g}^{\mathrm{MCT}-V}$	$s_{\mathrm{ex}}^{H}\left(\mathrm{MCT}-V\right)$	$s_{\mathrm{ex}}^{\mathrm{RT}}\left(\mathrm{MCT}-V\right)$
0.0	289.8	−5.204	−5.547	279.7	−5.124	−5.469
0.2	291.7	−5.188	−5.552	282.5	−5.115	−5.481
0.4	299.5	−5.161	−5.557	291.3	−5.098	−5.496
0.6	314.6	−5.134	−5.558	306.4	−5.074	−5.499
0.8	337.8	−5.111	−5.551	328.8	−5.05	−5.491
1.0	370.3	−5.093	−5.540	359.3	−5.025	−5.474
1.2	413.5	−5.062	−5.526	399.5	−4.984	−5.451
1.4	469.5	−5.082	−5.512	451.6	−4.992	−5.427
2.0	746.3	−5.002	−5.469	711.5	−4.893	−5.366
2.6	1308	−4.932	−5.444	1249	−4.827	−5.344
3.0	1980	−4.884	−5.436	1905	−4.798	−5.353
3.6	3871	−4.782	−5.441	3792	−4.737	−5.396
4.0	6210	−4.602	−5.453	6182	−4.593	−5.443
4.6	13,010	−4.614	−5.481	13,330	−4.662	−5.534
5.0	21,650	−4.626	−5.503	22,660	−4.715	−5.605
AVE		−4.695	−5.505		−4.912	−5.455
$\epsilon_{a}$ (%)		3.628	0.739		3.151	0.994
$\epsilon_{m}$ (%)		10.84	1.253		6.494	2.748

**Table 3:** Prospective vitrification indicators along the MCT-I glass transition line (columns 3 and 4) and the MCT-V glass transition line (columns 6 and 7). $S_{R2}^{\mathrm{APP}}$ denotes the amplitude ratio of the first nonzero minimum to the second maximum of the static structure factor obtained from approximation APP, while $g_{R2}^{\mathrm{APP}}$ denotes the amplitude ratio of the first nonzero minimum to the second maximum of the radial distribution function stemming from approximation APP. In the last three rows, the designation AVE denotes the average value of the vitrification indicator along the glass transition line (rows 1–15), while the quantities $\epsilon_{a}$ and $\epsilon_{m}$ report the average and maximum relative deviations between the vitrification indicators along the respective glass transition line and their average value reported in AVE.

κ	$\Gamma_{g}^{\mathrm{MCT}-I}$	$S_{R2}^{\mathrm{IEMHNC}}$	$g_{R2}^{\mathrm{IEMHNC}}$	$\Gamma_{g}^{\mathrm{MCT}-V}$	$S_{R2}^{\mathrm{VMHNC}}$	$g_{R2}^{\mathrm{VMHNC}}$
0.0	289.8	0.37	0.31	279.7	0.35	0.30
0.2	291.7	0.37	0.31	282.5	0.35	0.30
0.4	299.5	0.37	0.31	291.3	0.35	0.30
0.6	314.6	0.37	0.31	306.4	0.35	0.30
0.8	337.8	0.37	0.31	328.8	0.35	0.30
1.0	370.3	0.37	0.31	359.3	0.35	0.30
1.2	413.5	0.37	0.31	399.5	0.35	0.30
1.4	469.5	0.37	0.31	451.6	0.35	0.30
2.0	746.3	0.37	0.31	711.5	0.35	0.30
2.6	1308	0.37	0.31	1249	0.35	0.29
3.0	1980	0.37	0.31	1905	0.34	0.29
3.6	3871	0.36	0.31	3792	0.34	0.29
4.0	6210	0.36	0.31	6182	0.34	0.29
4.6	13,010	0.36	0.31	13,330	0.34	0.30
5.0	21,650	0.36	0.31	22,660	0.34	0.30
AVE		0.37	0.31		0.35	0.30
$\epsilon_{a}$ (%)		1.22	0.20		1.24	0.57
$\epsilon_{m}$ (%)		2.32	0.60		2.34	0.86

## Data Availability

The data presented in this study are available on request from the corresponding author.

## Abbreviations

The following abbreviations are used in this manuscript:

GMCT	Generalized Mode Coupling Theory
HNC	Hypernetted Chain
HS	Hard Sphere
HSPY	Hard Sphere Percus–Yevick
HSVW	Hard Sphere Verlet-Weis
IEMHNC	Isomorph-based Empirically Modified HNC
IET	Integral Equation Theory
IPL	Inverse Power Law
MCT	Mode Coupling Theory
MCT-H	MCT solved with structural input from the HNC approximation
MCT-I	MCT solved with structural input from the IEMHNC approximation
MCT-V	MCT solved with structural input from the VMHNC approximation
MD	Moleculary Dynamics
OCP	One-Component Plasma
PY	Percus–Yevick
YOCP	Yukawa One-Component Plasma
VMHNC	Variationally Modified HNC
